# The pacemaker-twiddler’s syndrome: an infrequent cause of pacemaker failure

**DOI:** 10.1186/s13104-015-1818-0

**Published:** 2016-01-20

**Authors:** Mohammad Salahuddin, Fathima Aaysha Cader, Sahela Nasrin, Mashhud Zia Chowdhury

**Affiliations:** Department of Cardiology, Ibrahim Cardiac Hospital and Research Institute (ICHRI), Dhaka, Bangladesh

**Keywords:** Twiddler’s syndrome, Pacemaker failure, Lead dislodgement

## Abstract

**Background:**

The pacemaker-twiddler’s syndrome is an uncommon cause of pacemaker malfunction. It occurs due to unintentional or deliberate manipulation of the pacemaker pulse generator within its skin pocket by the patient. This causes coiling of the lead and its dislodgement, resulting in failure of ventricular pacing. More commonly reported among elderly females with impaired cognition, the phenomenon usually occurs in the first year following pacemaker implantation. Treatment involves repositioning of the dislodged leads and suture fixation of the lead and pulse generator within its pocket.

**Case presentation:**

An 87 year old Bangladeshi lady who underwent a single chamber ventricular pacemaker (VVI mode: i.e. ventricle paced, ventricle sensed, inhibitory mode) implantation with the indication of complete heart block, and presented to us again 7 weeks later, with syncopal attacks. She admitted to repeatedly manipulating the pacemaker generator in her left pectoral region. Physical examination revealed a heart rate of 42 beats/minute, blood pressure 140/80 mmHg and bilateral crackles on lung auscultation. She had no cognitive deficit.

An immediate electrocardiogram showed complete heart block with pacemaker spikes and failure to capture. Chest X-ray showed coiled and retracted right ventricular lead and rotated pulse generator. An emergent temporary pace maker was set at a rate of 60 beats per minute. Subsequently, she underwent successful lead repositioning with strong counselling to avoid further twiddling.

**Conclusion:**

Twiddler’s syndrome should be considered as a cause of pacemaker failure in elderly patients presenting with bradyarrythmias following pacemaker implantation. Chest X-ray and electrocardiograms are simple and easily-available first line investigations for its diagnosis. Lead repositioning is required, however proper patient education and counselling against further manipulation is paramount to long-term management.

## Background

The pacemaker-twiddler’s syndrome refers to the permanent malfunction of a pacemaker resulting from manipulation of the pulse generator within its skin pocket [1]. This leads to a rotation of the device, coiling of the lead and its dislodgement, leading to pacemaker failure. First described by Bayliss in 1968, it is a rare but potentially fatal complication of pacemaker treatment [1]. We believe it is under-reported in a South Asian setting, and should be considered as a possible cause of pacemaker failure, particularly in elderly patients presenting with dizziness, syncope and bradyarrhythmias, post Permanent Pacemaker (PPM)-implantation.

We report the case of an 87 year old Bangladeshi lady who presented with recurrent syncopal attacks seven weeks after PPM implantation. She admitted to repeatedly twiddling with the PPM pulse generator. Electrocardiography (ECG) showed complete heart block with pacemaker spikes and failure to capture. Chest X-ray showed a rotated pulse generator and coiled and dislodged right ventricular (RV) lead. Pacemaker lead re-positioning was done and she was counseled against further twiddling of the generator.

## Case report

An 87 year old Bangladeshi lady presented with a 4 month history of recurrent episodes of syncope associated with complete loss of consciousness for several seconds, and shortness of breath. Her medical history was significant for ischaemic heart disease, hypertension, diabetes mellitus and chronic kidney disease. Physical examination revealed a heart rate of 42 beats/minute, blood pressure 140/80 mmHg, bilateral crackles on lung auscultation and auscultation of the praecordium revealed no abnormality. She had no cognitive deficit.

**Fig. 1:**
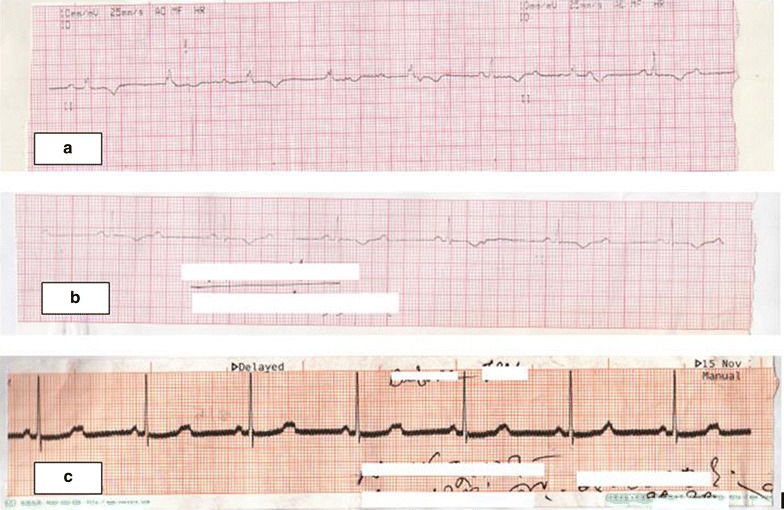
ECG tracing during initial presentation showing different arrhythmias. **a** Complete heart block, **b** 2:1 Atrio-ventricular block, **c** Sinus bradycardia

An immediate ECG revealed intermittent complete heart block alternating with junctional rhythm and sinus bradycardia, with a heart rate of approximately 42 beats/minute (Fig. 1). An emergent temporary pace maker (TPM) was set at a rate of 60 beats per minute. Echocardiogram revealed anterior wall hypokinesia with a left ventricular ejection fraction of 50 %. Routine laboratory parameters were within normal limit, except for an elevated serum creatinine of 2.28 mg/dL and urea of 120 mg/dL. Electrolyte imbalance and hypothyroidism were excluded biochemically. Drug history did not include beta blockers. She was treated with diuretics in addition to her anti-ischaemic treatment. The patient remained TPM dependent for the following 2 days.

She was diagnosed as a case of complete heart block. As this is a Class I indication for PPM implantation according to AHA/ACC/ESC guidelines, a single chamber ventricular pacemaker (VVI mode: i.e. Ventricle paced, Ventricle sensed, Inhibitory mode) was implanted with appropriate lead parameters for sensing and pacing. The pulse generator was positioned in the left pre-pectoral area, and the right ventricular lead was placed via the left subclavian vein.

The post-implantation chest X-ray showed ideal ventricular lead positioning, with the pacemaker head pointing upwards. Post PPM ECG showed regular ventricular paced rhythm. She had no further episodes of syncope and was discharged 3 days later with stable hemodynamic parameters.

**Fig. 2:**
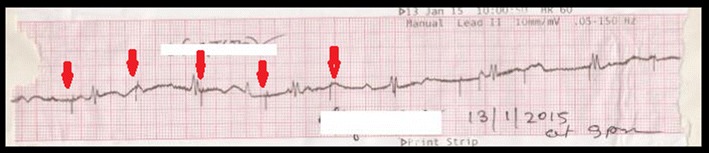
ECG tracing after Twiddler’s syndrome developed. *ECG tracing* showing complete heart block with pacemaker spikes (*red arrows*) and failure to capture

**Fig. 3:**
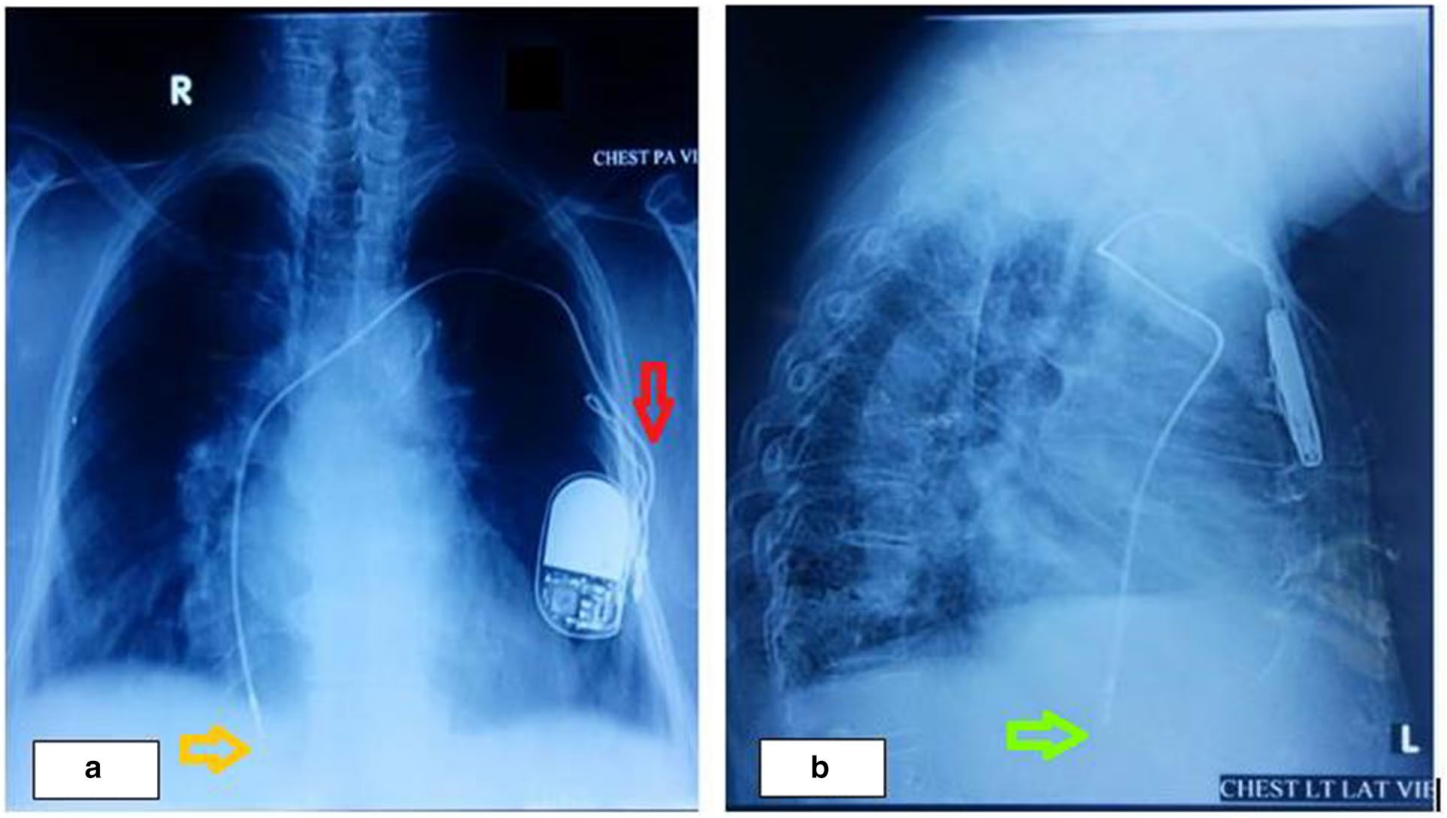
Chest X-ray demonstrating evidence of Twiddler’s syndrome. **a** Chest X-ray posterior–anterior view showing coiling of the proximal part of ventricular lead and rotated pulse generator with its head pointing downwards (*red arrow*) and displaced tip of the right ventricular lead (*orange arrow*). **b** Chest X-ray lateral view showing the distal end of the ventricular lead in the right atrium (*green arrow*)

Seven weeks after pacemaker implantation, she presented again with recurrent episodes of syncope. She had eight episodes of syncope in the 3 days prior to presentation, each lasting less than fifteen seconds, following which she regained consciousness. The ECG showed complete heart block with pacemaker spikes and failure to capture (Fig. 2). The chest X-ray showed coiling of the ventricular lead resulting in the pulse generator being rotated, with its head now pointing downwards. The distal end of the ventricular lead was seen in the right atrium (Fig. 3).

On query the patient admitted that she had repeatedly twiddled and manipulated the PPM generator in the skin pocket on her upper left chest, especially attempting to show the device to other elderly neighbours. She denied any history of epigastric or arm twitching. She was immediately put on TPM once again, and subsequently PPM lead repositioning was performed. During this procedure, the leads were found to be grossly entangled into repeated knots. They were uncoiled manually, and the pulse generator was secured in the pocket with additional non-absorbable sutures.

A chest X-ray was done to confirm appropriate lead positioning. She was discharged on day 3 with good counselling against further twiddling of the device. She has been asymptomatic since then.

## Discussion

Continuous ‘twiddling’ or manipulation of the pulse generator within its skin pocket, by the patient, leads to a painless dislodgment of device: subsequent coiling of the lead causes lead dislodgement, ultimately resulting pacemaker malfunction [1]. Variations of the phenomenon, leading to fatal device failure has also been reported with implantable cardioverter-defibrillators [2, 3] and cardiac resynchronisation therapy [3].

The pacemaker-twiddler’s syndrome has an estimated frequency of around 0.07–7 % [4–6]. The majority of cases are diagnosed within the first year of implant [2, 3], although it can occur at any time after device implantation. More recently a late Twiddler’s syndrome has also been described [7]. Our patient presented after 7 weeks of PPM implantation.

Manifestations of Twiddler’s syndrome vary, depending on the degree of entanglement, subsequent retraction of the electrode and final site of the dislodged lead. Leads that get dislodged further up may stimulate the ipsilateral phrenic nerves causing diaphragmatic contractions, occasional spasms of involuntary respiration or hiccups [1, 3, 4]. Further coiling and withdrawal of the lead, leads to stimulation of the brachial plexus, resulting in rhythmic arm twitching [1, 3, 4, 8]. Our patient did not experience these symptoms, as the retracted lead in our patient was confined to the right atrium.

The risk factors for the condition include female gender, obesity, elderly age group, impaired cognition and a smaller-sized implanted device relative to its pocket [4, 5, 9]. An associated increased laxity of the subcutaneous tissues, particularly in elderly patients facilitates further dislodgement of device [2, 3, 5]. Additionally, the smaller sizes of newer devices easily permit their rotation within the skin pocket [3.7].

The majority of patients with the syndrome deny manipulating the device [3, 4], although the syndrome encompasses deliberate manipulation as well [8, 9]. Our patient admitted to deliberate twiddling of the device, an unfortunate phenomenon which could possibly have been prevented with better patient education.

The chest X-ray is the simplest and most vital diagnostic tool to diagnose Twiddler’s syndrome [3], as it is rapid and gives a clear image of the lead coiling and device rotation. However, chest X-rays are frequently overlooked, and the focus is very often more directed at Holter monitoring. Admittedly, although Holter sheds light on the nature of the arrhythmia, the exact pinpointing of the cause of pacemaker failure in such cases, cannot be reached without X-ray imaging.

Treatment of diagnosed cases include uncoiling of the lead (done in our patient), implantation of a new lead and repositioning of the pulse generator [3, 8, 9]. As lax subcutaneous tissues permit device rotation, minimizing the pocket size, and suture fixation of the pulse generator with a ligature during implantation can prevent the occurrence of Twiddler’s syndrome [3–5, 7, 8].

A smaller and tightly fitting pocket without redundant space around the generator could also achieve better device fixation [5, 7]. Some authors advocate active fixation of the transvenous leads with non-absorbable suture [1, 4] or the use of a Dacron patch to promote tissue growth around the device and promote better fixation [1, 4, 10]. In their article, Fahraeus and Hoijer reserved the option of suturing the device to the fascia for patients with mental disorders, confusion and lax subcutaneous tissue [5].

Despite more stringent suturing procedures, proper patient education and counselling to care-givers, especially in elderly patients, remains the single most important means for avoiding PPM manipulations and preventing such fatal consequences [3].

## Conclusions

Twiddler’s syndrome should be considered as a cause of pacemaker failure, particularly in elderly patients presenting with bradyarrythmias following pacemaker implantation. Chest X-ray is a simple and easily-available investigation that will rapidly clinch the diagnosis, in order to initiate early rectification of the problem. Proper patient education and counselling against manipulating the pacemaker generator is the single-most important preventive strategy.

## Consent

Written informed consent was obtained from the patient for publication of this case report and any accompanying images.

## Abbreviations

PPM: permanent pacemaker
ECG: electrocardiogram
RV: right ventricle
TPM: temporary pacemaker

## References

[1] Bayliss CE, Beanlands DS, Baird RJ (1968). The pacemaker-twiddler’s syndrome: a new complication of implantable transvenous pacemakers. Can Med Assoc 0.0J.

[2] Sharifi M, Inbar S, Neckels B, Shook H (2005). Twiddling to the extreme: development of twiddler syndrome in an implanted cardioverter-defibrillator. J Invasive Cardiol.

[3] DeMarco DC, Xuereb RG (2009). ‘Twiddling’ of the pacemaker resulting in lead dislodgement. Malta Med J.

[4] Mandal M, Pande A, Kahali D (2012). A rare case of very early pacemaker twiddler’s syndrome. Heart Views.

[5] Fahraeus T, Hijer CJ (2003). Early pacemaker twiddler syndrome. Europace.

[6] Hill PE (1987). Complications of permanent transvenous cardiac pacing: a 14-year review of all transvenous pacemakers inserted at one community hospital. Pacing Clin Electrophysiol.

[7] Dursun I, Yesildag O, Soylu K, Yilmaz O, Yasar E, Meric M (2006). Late pacemaker twiddler syndrome. Clin Res Cardiol.

[8] Nicholson WJ, Tuohy KA, Tilkemeier P (2003). Twiddler’s syndrome. N Engl J Med.

[9] Castilo R, Cavusoglu E (2006). Twiddler’s syndrome: an interesting cause of pacemaker failure. Cardiology.

[10] Furman S (1995). Defibrillator Twiddler’s syndrome. Ann Thorac Surg.

